# Enhanced Thermoelectric Cooling through Introduction of Material Anisotropy in Transverse Thermoelectric Composites

**DOI:** 10.3390/ma12132049

**Published:** 2019-06-26

**Authors:** Bosen Qian, Fei Ren, Yao Zhao, Fan Wu, Tiantian Wang

**Affiliations:** 1 Key Laboratory of Traffic Safety on Track, Ministry of Education, School of Traffic and Transportation Engineering, Central South University, Changsha 410075, China; 2 Department of Mechanical Engineering, Temple University, Philadelphia, PA 19122, USA; 3 Joint International Research Laboratory of Key Technology for Rail Traffic Safety, School of Traffic and Transportation Engineering, Central South University, Changsha 410075, China; 4 National and Local Joint Engineering Research Center of Safety Technology for Rail Vehicle, School of Traffic and Transportation Engineering, Central South University, Changsha 410075, China

**Keywords:** thermoelectric cooling, transverse thermoelectricity, figure of merit, composite material, material anisotropy

## Abstract

Transverse thermoelectric materials can achieve appreciable cooling power with minimal space requirement. Among all types of material candidates for transverse thermoelectric applications, composite materials have the best cooling performance. In this study, anisotropic material properties were applied to the component phase of transverse thermoelectric composites. A mathematical model was established for predicting the performance of fibrous transverse thermoelectric composites with anisotropic components. The mathematical model was then validated by finite element analysis. The thermoelectric performance of three types of composites are presented, each with the same set of component materials. For each type of component, both anisotropic single-crystal and isotropic polycrystal material properties were applied. The results showed that the cooling capacity of the system was improved by introducing material anisotropy in the component phase of composite. The results also indicated that the orientation of the anisotropic component’s property axis, the anisotropic characteristic of a material, will significantly influence the thermoelectric performance of the composite. For a composite material consisting of Copper fiber and Bi_2_Te_3_ matrix, the maximum cooling capacity can vary as much as 50% at 300 K depending on the property axis alignment of Bi_2_Te_3_ in the composite. The composite with Copper and anisotropic SnSe single crystal had a 51% improvement in the maximum cooling capacity compared to the composite made of Copper and isotropic SnSe polycrystals.

## 1. Introduction

Thermoelectric material can transform thermal energy into electrical energy and vice versa. Its applications can be found in various fields such as temperature measurement, power generation, temperature control, etc. Conventional thermoelectric devices consist of both n-type and p-type thermoelectric legs. Within each leg, the heat flux and electrical current flow are parallel to each other [1,2]. Transverse thermoelectric devices make use of the transverse Seebeck effect so that the electrical current and heat flux flow perpendicular to each other [3]. There are four main types of transverse thermoelectric materials, which are anisotropic single-crystal material, polycrystal material with engineered anisotropy, anisotropic organic thin-film thermoelectrics, and anisotropic thermoelectric composites. The single-crystal material anisotropy is the result of an unsymmetrical lattice structure [4]; the material anisotropy in polycrystal is contributed to by both the lattice structure of the grain crystal and the grain geometry [5]. The organic materials anisotropic property is caused by the preferred alignment of polymer chains [6,7,8]. Studies have shown that the anisotropic thermoelectric composites can provide the best system efficiency among all of the candidates [9]. 

The transverse thermoelectric composite can further be categorized into layered and fibrous thermoelectric composites. These two types of composites can also be seen as two-dimensional (2D) and one-dimensional (1D) inclusion composites. For layered composites with isotropic components, Babin et al. [10] established a mathematical model for fast prediction of dimensionless transverse thermoelectric figure of merit (Z_trans_T), while other researchers performed finite element simulations and experimental tests and validated such a mathematical model [11,12,13,14,15]. Similar to layered composites, fibrous composites can also provide appreciable Z_trans_T values. A mathematical model was established by Qian [16] to study the thermoelectric performance of fibrous composites.

Until now, most studies on transverse thermoelectric composites assumed isotropic material properties in the component phase. However, many studies have already shown that thermoelectric materials can exhibit anisotropic properties, such as Bi_2_Te_3_ [5,17], SnSe [18], and organic thermoelectric PEDOT:PSS (poly(3,4-ethylenedioxythiophene) polystyrene sulfonate) [19,20], etc. As a result, anisotropic material properties were applied into the component phase of layered transverse thermoelectric composites, and the results showed that the maximum Z_trans_T could be improved by introducing material anisotropy in a polycrystal [21]. However, the effect of material anisotropy on fibrous composite still remains unknown.

In this study, anisotropic material properties were applied to the component phase of fibrous transverse thermoelectric composites. A mathematical model was established for predicting the effective material properties and thermoelectric cooling capacities of the composite. Finite element simulations were conducted to validate the mathematical model. A case study was conducted on a fibrous composite material containing Copper fibers and an anisotropic Bi_2_Te_3_ matrix, where the possible aggregate (anisotropic) properties for Bi_2_Te_3_ were calculated and applied into the component phase. The influence of the material anisotropy and the material property axis alignment in the composite were then discussed with respect to the maximum Z_trans_T and maximum cooling capacity of the composites. In the next step, the cooling performances of both the layered and the fibrous composites were compared. The thermoelectric properties of three representative composites, which were Bi_2_Te_3_/Copper, In_4_Se_2.25_/Copper, and SnSe/Copper, were applied into the component phase of the composites under different material property axis alignment configurations and extreme aggregate properties of the anisotropic component. The influence of material anisotropy on the enhancement of the maximum cooling capacity and maximum Z_trans_T of the transverse thermoelectric composites was demonstrated. The cooling capacities of the layered and fibrous composites were also compared. The derived mathematical model can serve as an efficient tool for selecting high-performance fibrous transverse thermoelectric composites, while the comparison between fibrous and layered thermoelectric composites can inspire and assist thermoelectric researchers in designing higher performance thermoelectric devices.

## 2. Mathematical Model for Fibrous Thermoelectric Composites with Anisotropic Components 

**Figure 1:**
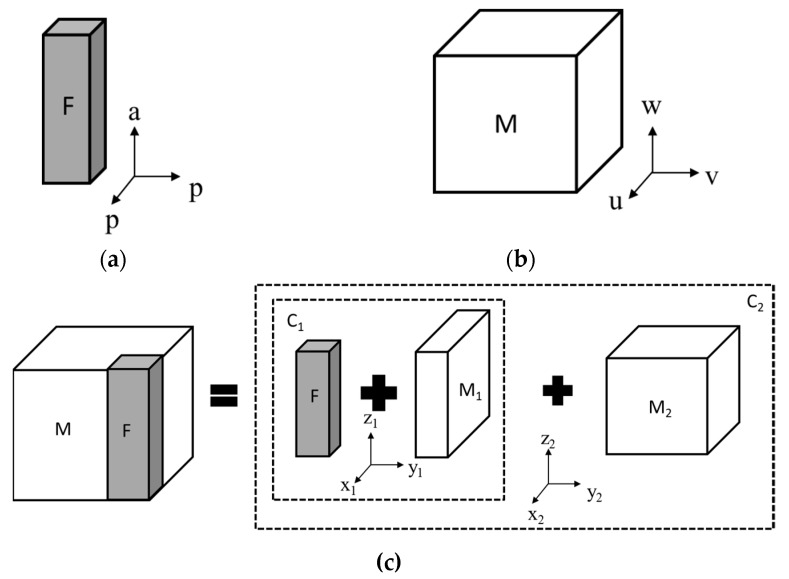
Schematic of the unit cell model in the fibrous thermoelectric composite which consisted of a fibrous (F) and matrix (M) phase. (**a**) Material property coordinate system of the fibrous phase; (**b**) material property coordinate system of the matrix phase; (**c**) schematic of the unit cell structure.

In this section, analytical equations are derived for the effective properties of a transverse thermoelectric composite. In the mathematical model, unit cell structure is used to reduce the complexity of this problem. This unit cell model has been proven to be a convenient tool in studies on the effective properties of a material with periodical structures [16,22]. The schematic of a fibrous thermoelectric composite unit cell is shown in Figure 1, where a fiber (F) with a square cross-section was placed at the corner of a cubic matrix (M). The square fiber was used in the unit cell model for mathematical simplicity. The contact between the matrix and fibrous material was assumed to be ideal so that electrical and thermal contact resistance were not considered.

When anisotropic material properties are introduced into the component phase (i.e., fibrous phase and matrix phase) of a composite material, the alignment of the material local property axis with respect to the composite material coordinate system plays a key role in the effective properties of the composite. Many 1D inclusions, such as carbon nanotubes [23,24], have different material properties along and perpendicular to the fiber axis direction. Therefore, in this study, we used the subscript “p” to represent the properties on the cross-sectional plane of the fiber and subscript “a” to represent properties along fiber axis (Figure 1a). As for the matrix phase, we used the material local axis system “uvw” to describe the anisotropic material properties (Figure 1b). There are four sets of coordinate systems in Figure 1, which are the local material coordinate system of the fibrous and matrix phase, the coordinate system of composite C_1_(F + M_1_), and the coordinate system of unit cell C_2_(F + M_1_ + M_2_). In Figure 1, the coordinate axes u, x_1_, and x_2_ align parallel to each other; v, y_1_, and y_2_ align parallel to each other, and a, w, z_1_, and z_2_ align parallel to each other. The cross-sectional plane of fiber (p–p) is parallel with the u–v plane of the matrix material.

The effective properties of the unit cell can be derived in two steps as illustrated in Figure 1c. In the first step, the square fiber (F) is combined with part of the matrix phase (M_1_) to form a rectangular composite C_1_, where the matrix block has the same width and height as the fibrous phase. In the second step, the composite C_1_ is combined with the rest of the matrix to form the unit cell. The effective thermal and electrical conducting properties of the unit cell can be calculated using Kirchhoff’s law, and the effective Seebeck coefficient can be calculated using Thevenin’s theorem [25]. The material properties of the composite C_1_ can be derived according to Equation (1):

(1) $$\begin{array}{ccc} \rho_{x_{1}}=\frac{n\rho_{M_{u}}+\rho_{F_{p}}}{1+n} & \lambda_{x_{1}}=\frac{\lambda_{F_{p}}\lambda_{M_{u}}\left(1+n\right)}{n\lambda_{F_{p}}+\lambda_{M_{u}}} & S_{x_{1}}=\frac{S_{F_{p}}\lambda_{M_{u}}+nS_{M_{u}}\lambda_{F_{p}}}{n\lambda_{F_{p}}+\lambda_{M_{u}}}\\ \rho_{y_{1}}=\frac{\rho_{M_{v}}\rho_{F_{p}}\left(1+n\right)}{\rho_{M_{v}}+n\rho_{F_{p}}} & \lambda_{y_{1}}=\frac{\lambda_{F_{p}}+n\lambda_{M_{v}}}{1+n} & S_{y_{1}}=\frac{S_{F_{p}}\rho_{M_{v}}+nS_{M_{v}}\lambda_{F_{p}}}{n\rho_{F_{p}}+\rho_{M_{v}}}\\ \rho_{z_{1}}=\frac{\rho_{M_{w}}\rho_{F_{a}}\left(1+n\right)}{\rho_{M_{w}}+n\rho_{F_{a}}} & \lambda_{z_{1}}=\frac{\lambda_{F_{a}}+n\lambda_{M_{w}}}{1+n} & S_{z_{1}}=\frac{S_{F_{a}}\rho_{M_{w}}+nS_{M_{w}}\lambda_{F_{a}}}{n\rho_{F_{a}}+\rho_{M_{w}}} \end{array}$$

The symbols *ρ*, *λ*, *S*, and *f* stand for electrical resistivity, thermal conductivity, Seebeck coefficient, and fiber volume fraction. The subscripts p, a, u, v, w, x_i_, y_i_, and z_i_ stand for component material’s properties along each material’s local coordinate systems. The material properties of the unit cell can be derived according to Equation (2):

(2) $$\begin{array}{ccc} \rho_{x_{2}}=\frac{\rho_{x_{1}}\rho_{M_{u}}}{\sqrt{f}\left(\rho_{M_{u}}-\rho_{x_{1}}\right)+\rho_{x_{1}}} & \lambda_{x_{2}}=\left(\lambda_{x_{1}}+\left(\frac{1}{\sqrt{f}}-1\right)\;\lambda_{M_{u}}\right)\sqrt{f} & S_{x_{2}}=\frac{S_{x_{1}}\rho_{M_{u}}+\left(\frac{1}{\sqrt{f}}-1\right)S_{M_{u}}\rho_{x_{1}}}{\left(\frac{1}{\sqrt{f}}-1\right)\rho_{x_{1}}+\rho_{M_{u}}}\\ \rho_{y_{2}}=\sqrt{f}\left(\rho_{y_{1}}-\rho_{v}\right)+\rho_{M_{v}} & \lambda_{y_{2}}=\frac{\lambda_{y_{1}}\lambda_{M_{v}}}{\sqrt{f}\left(\left(\frac{1}{\sqrt{f}}-1\right)\lambda_{y_{1}}+\lambda_{M_{v}}\right)} & S_{y_{2}}=\frac{S_{y_{1}}\lambda_{M_{v}}+\left(\frac{1}{\sqrt{f}}-1\right)S_{M_{v}}\lambda_{y_{1}}}{\left(\frac{1}{\sqrt{f}}-1\right)\lambda_{y_{1}}+\lambda_{M_{v}}}\\ \rho_{z_{2}}=\frac{\rho_{z_{1}}\rho_{M_{w}}}{\sqrt{f}\left(\rho_{M_{w}}-\rho_{z_{1}}\right)+\rho_{z_{1}}} & \lambda_{z_{2}}=\left(\lambda_{z_{1}}+\left(\frac{1}{\sqrt{f}}-1\right)\;\lambda_{M_{w}}\right)\sqrt{f} & S_{z_{2}}=\frac{S_{z_{1}}\rho_{M_{w}}+\left(\frac{1}{\sqrt{f}}-1\right)S_{M_{w}}\rho_{z_{1}}}{\left(\frac{1}{\sqrt{f}}-1\right)\rho_{z_{1}}+\rho_{M_{w}}} \end{array}$$

**Figure 2:**
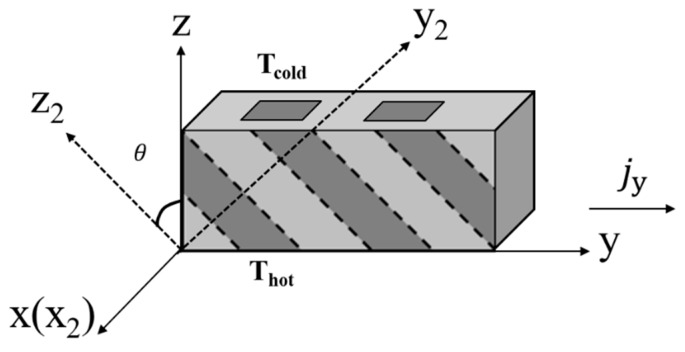
Schematic of a fibrous transverse thermoelectric composite with tilted fibers.

In Figure 1, the fiber axis aligns parallel to the z_2_-axis of the composite. When fibers are tilted aligned in the composite, the effective properties of the composite can be calculated through matrix transformation. For example, if the fibers in Figure 1 are rotated by an angle of *θ* around x_2_-axis into the configuration in Figure 2, the effective properties of the composite can be calculated as [26]:

(3) $$P_{xyz}=\left[\begin{array}{ccc} P_{x_{2}} & 0 & 0\\ 0 & P_{y_{2}}cos^{2}\left(\theta \right)+P_{z_{2}}sin^{2}\left(\theta \right) & \frac{1}{2}\left(P_{z_{2}}-P_{y_{2}}\right)sin\left(2\theta \right)\\ 0 & \frac{1}{2}\left(P_{z_{2}}-P_{y_{2}}\right)sin\left(2\theta \right) & P_{z_{2}}cos^{2}\left(\theta \right)+P_{y_{2}}sin^{2}\left(\theta \right) \end{array}\right]$$

In a transverse thermoelectric material, heat flux and electrical current flow perpendicular to each other. Based on Figure 2, we assume the electrical current flows in the y-direction and heat flux flows in the z-direction. The transverse thermoelectric figure of merit is defined as $Z_{\mathrm{trans}}T=\frac{S_{zy}^{2}T}{\rho_{yy}\lambda_{zz}}$ [10,27], where *S_zy_* is the transverse Seebeck coefficient, *ρ_yy_* is electrical resistivity in the y-direction, *λ_zz_* is thermal conductivity in the z-direction, T is the operating temperature. The term *S_zy_*, *ρ_yy_*, and *λ_zz_* values can be calculated using Equation (1) to Equation (3).

Figure 2 can also represent a fibrous transverse thermoelectric device operating under cooling mode, where the top surface serves as a cooling surface with a temperature of T_c_ and the bottom surface serves as a heat sink with a temperature of T_h_. When the electrical current flows in the y-direction, the transverse Peltier effect will trigger heat flux along the z-direction. The maximum cooling capacity of the system is related to the Z_trans_T value, according to previous studies [10,28]. In this study, the T_h_ was set to be the measuring temperature of material properties, which are shown in Table 1.

In the next step, experimentally measured material properties are implemented into the derived equations. Previous studies [9] have shown that high-performance transverse thermoelectric composites usually consist of one semiconducting thermoelectric phase and one highly conductive phase, where the thermoelectric phase provides high Seebeck coefficient and low thermal conductivity, the conducting phase provides low electrical resistivity. Therefore, we chose anisotropic Bi_2_Te_3_ polycrystal as the matrix phase and Copper as the fibrous phase for the composite. The properties of these materials are shown in Table 1. According to Table 1, the Copper phase was isotropic and Bi_2_Te_3_ was anisotropic with planar (u–v plane) isotropy. 

**Figure 3:**
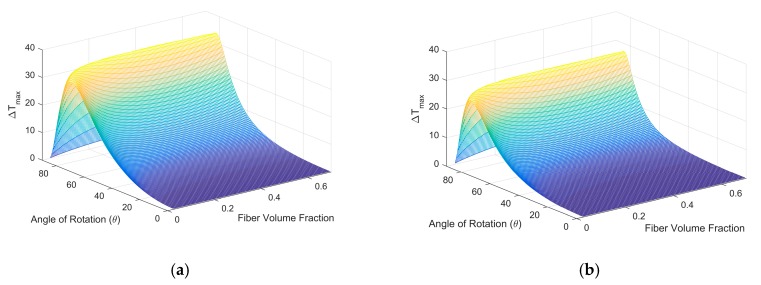
Maximum cooling capacity (∆T_max_) of the Bi_2_Te_3_ matrix/Cu fiber composite at 300 K under different material local coordinate system alignment configurations. (**a**) Configuration I; (**b**) Configuration II.

When anisotropic material properties are implemented in the component phase of a composite, the alignment of component material’s local property coordinate system, with respect to the composite coordinate system, will affect the effective properties of the composite. In Figure 1, the u–v plane of the matrix material aligns perpendicular to the fiber axis. If the material property coordinate in Figure 1b is rotated around the u-axis 90 degrees, the anisotropy plane of Bi_2_Te_3_ will be parallel to the fiber axis direction. For the convenience of analysis, we shall refer to the two configurations mentioned above as configuration I and configuration II. In configuration I, the material property axes u, v, and w are parallel to the composite axes x_2_, y_2_, and z_2_, respectively. In configuration II, the material property axes u, v, and w are parallel to the composite axes x_2_, z_2_, and y_2_, respectively. The corresponding Z_trans_T and maximum cooling capacities (∆T_max_) based on these two configurations were calculated using the derived mathematical model with respect to fiber rotation angle, *θ*, and fiber volume fraction, *f*. The results are shown in Figure 3.

Based on Figure 3, the Z_trans_T and ∆T_max_ values exhibited similar trends for both configurations. The maximum values appearred at rotation angles between 75 and 85 degrees. The change in fiber volume fraction under a constant rotation angle had a minor influence on the Z_trans_T and ∆T_max_ values. The maximum Z_trans_T and ∆T_max_ values in configuration I were 0.29 and 34 K, while the values of maximum Z_trans_T and ∆T_max_ in configuration II were 0.22 and 27 K. These results indicate a 26% difference in the peak thermoelectric performance among the two configurations. Hence, the alignment of the material’s local property coordinates with respect to the composite coordinate system had significant influence on the composite’s cooling performance. 

**Figure 4:**
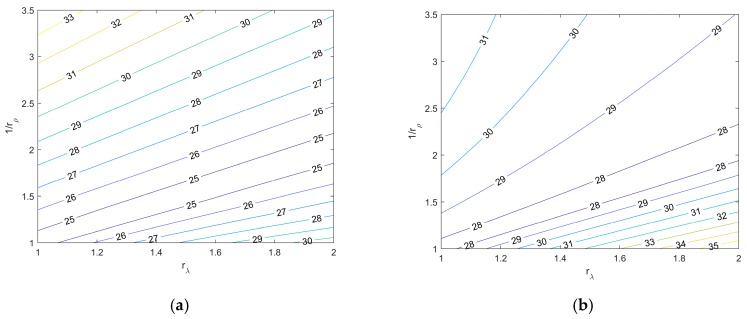
Maximum cooling capacity (∆T_max_) of Copper fiber/Bi_2_Te_3_ matrix transverse thermoelectric composite as a function of material anisotropy ratio ($r_{\lambda }$,$\;r_{\rho }$ ) of the Bi_2_Te_3_ phase. The aggregated values of the Bi_2_Te_3_ phase were calculated from single-crystal data (Table 1) using the (**a**) Reuss model and (**b**) Voigt model.

Many thermoelectric materials are polycrystals. These polycrystals may exhibit certain degrees of texture, and thus, anisotropic properties as a result of the fabrication processes. There exists theoretical models that can correlate the aggregate properties of polycrystals with its single-crystal material property. Among these theoretical models, the Voigt model and Reuss model can provide upper and lower bounds for the aggregate properties of polycrystals, respectively [32,33,34]. In this study, we take experimentally measured values from a single-crystal Bi_2_Te_3_ (Table 1) and calculate possible aggregate material properties using Reuss and Voigt models. The Bi_2_Te_3_ polycrystal has isotropic Seebeck coefficients and exhibits planar isotropy (u–v plane) in electrical resistivity and thermal conductivity based on existing experimental results [5,18,35,36]. The anisotropy ratio terms $r_{\lambda }$ and $\;r_{\rho }$ are used to relate the material properties in different axis directions, such that $r_{\lambda }=\lambda_{u}/\lambda_{w}$**,**$\;r_{\rho }=\rho_{w}/\rho_{u}$. By importing these hypothetical aggregate properties into the Bi_2_Te_3_ phase, the maximum cooling capacities were calculated for the Bi_2_Te_3_ matrix/Cu fiber composite. The results are shown in Figure 4. According to the definition of $r_{\lambda }$ and $\;r_{\rho }$, the bottom-left corner of Figure 4a,b represents the maximum cooling capacity of a polycrystal Bi_2_Te_3_ with isotropic material properties. The results in Figure 4 show that by introducing material anisotropy in a fibrous transverse thermoelectric composite, the cooling capacity of the composite can be improved by 7% (Figure 4b, Voigt model) and 21% (Figure 4a, Reuss model).

## 3. Finite Element Simulation for Fibrous Thermoelectric Composite with Anisotropic Components

In order to validate the effectiveness of the mathematical model, finite element simulations were carried out using COMSOL (COMSOL Multiphysics, COMSOL Inc., Burlington, MA, USA). A unit cell model based on Figure 1c was constructed using polycrystal Bi_2_Te_3_ as the matrix phase and Copper as the fiber phase assuming the isotropy plane of Bi_2_Te_3_ was perpendicular to the fiber axis. For all finite element models involved in this study, mesh convergence studies were carried out and the convergence error was limited to below 3%.

**Figure 5:**
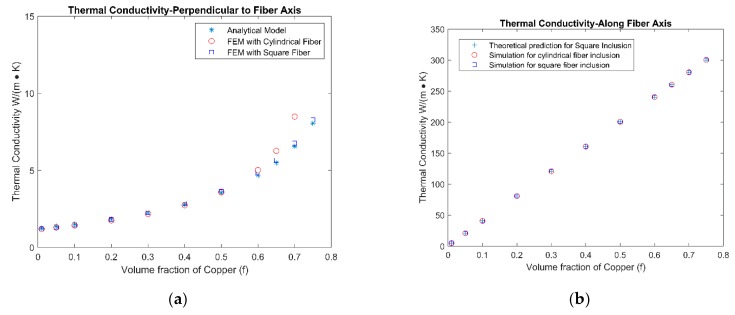

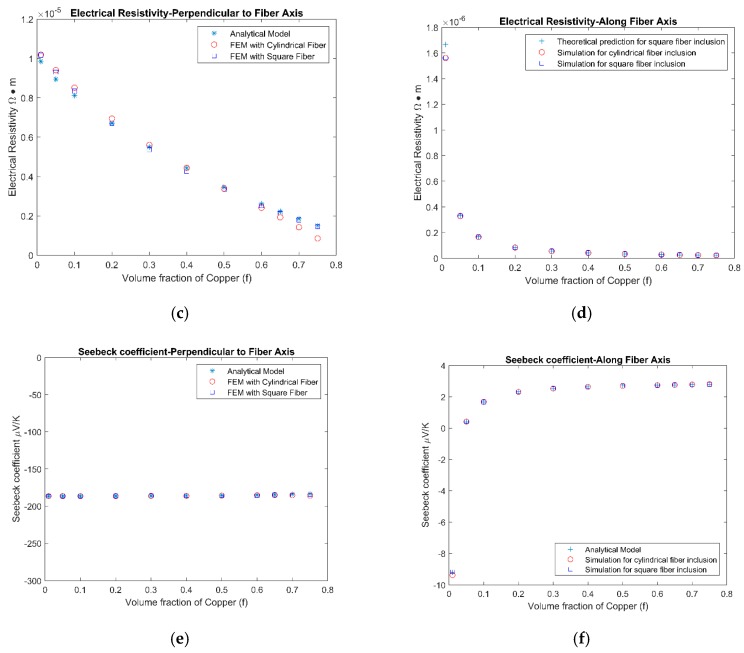
Finite element analysis results on effective properties of the unit cell (Figure 1c) in directions parallel and perpendicular to the fiber axis. (**a**) Thermal conductivity-Perpendicular to fiber axis; (**b**) Thermal conductivity-Along fiber axis; (**c**) Electrical Resistivity-Perpendicular to fiber axis; (**d**) Electrical Resistivity -Along fiber axis; (**e**) Seebeck coefficient-Perpendicular to fiber axis; (**f**) Seebeck coefficient-Along fiber axis.

According to the unit cell model shown in Figure 1c, square fiber was used for mathematical simplicity. In the finite element simulations, both cylindrical fiber and square fiber were investigated so as to explore the effect of fiber shapes. The effective properties of the unit cells are shown in Figure 5.

According to Figure 5, the finite element simulation results and mathematical model agreed well with each other. The conductivities of the unit cell increased as the volume fraction of the fibers increased, since the fiber was more conductive than the matrix material. In Figure 5f, the effective Seebeck coefficient had negative values at a low fiber volume fraction, but became positive as the fiber volume fraction increased. This was because the Bi_2_Te_3_ phase had a negative Seebeck coefficient and Copper had a positive Seebeck coefficient. The effective Seebeck coefficient was dominated by the matrix phase at low fiber volume fractions, and by the fibrous phase at high fiber volume fractions. 

Some minor deviations were observed between the mathematical model and the finite element simulation model. This was because the mathematical model assumes a one-dimensional flux flow, i.e., the electrical and heat flux only flow along the applied field, whereas fluxes in other directions are not considered. These fluxes are caused by the material inhomogeneity and the shape feature of fibers. When the fiber volume fraction is very small or very large, homogeneity of the composite will inevitably be compromised. Moreover, when the direction of the applied potential is not perpendicular to the interface between the matrix and fibrous phase, the secondary dimensional flux will occur. Both situations above are the cause of deviation between the mathematical model and finite element simulation results.

Finite element models with tilted aligned fibers in a matrix were also constructed to study the cooling capacity of the composite. The properties of polycrystal Bi_2_Te_3_ and Copper were applied. The fiber rotation angle was fixed at 80 degrees. According to Figure 2, the composite can be seen as fiber arrays periodically aligned in the x-direction. Therefore, to simplify the finite element model, only one fiber array was constructed inside the matrix block, and periodic boundary conditions were applied on the surfaces parallel to the y-z plane. The geometry of the model was 2 mm × 500 mm × 10 mm, while the fiber diameter was adjusted to suit different fiber volume fractions. The bottom surface of the device was fixed at 300 K to serve as a heat sink, while the top surface was subjected to natural convective heat transfer. Two 1 mm thick Copper blocks were added on both ends of the y-direction of the composite to serve as electrodes. Twenty cases were studied with different fiber volume fractions, fiber shapes, and with respect to the two configurations mentioned above. Within each case, the input electrical current density was adjusted until the maximum temperature difference between the top and bottom surface was reached.

**Figure 6:**
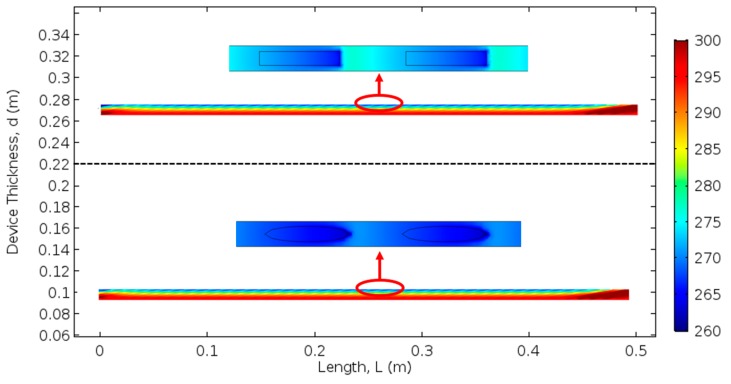
Temperature contour of fibrous transverse thermoelectric under cooling application. Upper: composite with square fibers. Lower: Composite with cylindrical fibers. Insect: Enlarged view of the composite’s cooling surface.

Figure 6 provides temperature distributions on the y–z plane for both cylindrical fiber model and square fiber model at 30% fiber volume fraction. Enlarged views of the top surface are shown in the insets. For both composites in Figure 6, a temperature gradient caused by the transverse Peltier effect can be clearly observed in the vertical direction. In the horizontal direction, the temperature was uniformly distributed except at the ends of the electrodes. In the insets of Figure 6, the abrupt temperature change at the interfaces of the fiber and matrix phases was the result of the Peltier effect. 

The surface temperature was averaged on the top surface of each finite element model. The maximum temperature difference in each case was calculated by subtracting the heat sink temperature by the averaged cooling surface temperature. The results are presented in Table 2. In general, the mathematical model results agreed well with the finite element simulation results with at most a 10% difference. These small discrepancies were mainly caused by the secondary dimensional flux that was previously discussed. The only exception in Table 2 was the case for the cylindrical fiber with f = 0.1 under configuration I, where the ∆T_max_ calculated using the mathematical model was more than 10% higher than the finite element simulation result. This was because under small or large fiber volume fraction cases, the homogeneity of the composite was very poor, which conflicts with the assumption in the mathematical model. The differences between the two configurations also agreed with the results shown in Figure 3, which emphasized the significant effect of the component material’s local property axes alignment in the composite coordinate system. During the finite element analysis, it was found that the size of the fibers has a slight influence on the TE performance. In anisotropic thermoelectric composites, smaller fibers provide the composite with better homogeneity. Although, under the same volume fraction of fibers the cooling capacity of the composite may not vary much with the size of the fibers, large fiber geometry is likely to cause higher temperature fluctuations in between the boundaries of the fibrous phase and the matrix phase.

## 4. Cooling Capacity Comparison for 1-Dimensional and 2-Demensional Inclusion Thermoelectric Composites with Anisotropic Components

The cooling performance of fibrous transverse thermoelectric composites were investigated with both a mathematical approach and a simulation approach. While the fibrous transverse thermoelectric composite can be treated as a 1D inclusion composite, the layered transverse thermoelectric composite can be treated as a 2D inclusion composite. The cooling capacity of the layered transverse thermoelectric composites with anisotropic components have been investigated thoroughly in previous studies [21]. Hence, it is informative to compare the cooling performances of transverse thermoelectric composites between 1D and 2D inclusion composites. In this section, three types of anisotropic thermoelectric crystals were chosen as the semiconducting phase of the composite, while Copper was chosen as the conducting phase of the composite. The material properties are listed in Table 1. The thermoelectric properties of Bi_2_Te_3_ single crystal were measured at 300 K, the thermoelectric properties of In_4_Se_2.25_ single crystal were measured at 600 K, and the thermoelectric properties of SnSe single crystal were measured at 700 K. The Copper served as the fibrous phase in the fibrous composite thermoelectric materials; its thermoelectric properties at 600 K and 700 K are also given in Table 1. It should be noticed that, in transverse thermoelectric composites, the transverse ZT matrix is anisotropic. The study made use of engineered anisotropy in composite materials and optimized the transverse ZT with respect to one specific element in the anisotropic ZT matrix. The anisotropic ZT values for other elements in the matrix are not discussed.

The semiconducting materials used in this comparison, i.e., Bi_2_Te_3_, In_4_Se_2.25_, and SnSe, all belong to planar isotropic material. Therefore, for both 1D and 2D inclusion composites consisting of semiconducting crystal and isotropic Copper, two representative configurations were used. Assuming the local material property axis as u_i_v_i_w_i_ (where the subscript i differentiates the type of materials), and the isotropy plane of material lies in the u_i_v_i_ plane. For fibrous composite materials, the definition of the two configurations were explained in previous sections of this study. For layered composite materials, in configuration III, the u_i_v_i_ plane aligns parallel to the layered plane of materials; in configuration IV, the u_i_v_i_ plane aligns perpendicular to the planar surface of the layers. Besides the four configurations, the conducting properties of isotropic polycrystal Bi_2_Te_3_ and SnSe, listed in Table 1, can be calculated using Reuss and Voigt models. Since Bi_2_Te_3_ and SnSe single crystals have isotropic Seebeck properties, their isotropic polycrystal Seebeck properties are the same as anisotropic single crystals. The conducting properties of the isotropic polycrystals are the average of the results calculated by the Reuss and Voigt models, which is referred as ‘Average RV’ in Table 3, respectively. 

The results in Table 3 present both maximum Z_trans_T as well as the maximum cooling capacity (∆T_max_) for composites with different anisotropic components and configurations using the derived mathematical model. It can be seen that, for composites which consist of the same combination of component materials but under different configurations, the alignment of component material’s property axis in the composite has a major influence on Z_trans_T as well as the ∆T_max_ values. For Bi_2_Te_3_/Copper composite, the difference in thermoelectric performances caused by the alignment of the anisotropic component’s property axis was 50%. For the SnSe/Copper composite, this difference exceeded 400%. This difference was caused by the variance of properties among different material property axes in the anisotropic component phase, where larger variance usually leads to bigger variance in the Z_trans_T and ∆T_max_ values of a composite.

When the cooling performance of the transverse thermoelectric was compared under the same component combination but with different inclusion types, it was found that for the composite with the same component volume fraction, the fibrous and layered composites yielded very similar ∆T_max_ as well as Z_trans_T values. However, it should be noted that the peak performances of the layered and fibrous composites under the same volume fraction were not identical to each other. The ∆T_max_ values were rounded up to the nearest integer, and the maximum Z_trans_T values were rounded up to the second decimal in Table 3. Although according to Table 3, the Z_trans_T and ∆T_max_ values in between different configurations have same values, it should be noted that none of the results in Table 3 are exactly identical to one another.

By comparing the results between the composite with anisotropic component material and isotropic component material, it was found that the anisotropic component in single-crystal form in both the layered transverse thermoelectric composite and the fibrous transverse thermoelectric composite can provide a better cooling performance compared to their isotropic polycrystal counterpart. For the Bi_2_Te_3_/Copper composite at 300 K, the composite with the anisotropic Bi_2_Te_3_ single crystal improved the maximum Z_trans_T and ∆T_max_ by as much as 15% compared to the composite with the isotropic Bi_2_Te_3_ polycrystal. For the SnSe/Copper composite at 700 K, the improvement in the maximum Z_trans_T and ∆T_max_ for composite with SnSe single crystal component was as much as 51% compared to composite with isotropic SnSe polycrystal components. For the In_4_Se_2.25_/Copper composite, the single-crystal form of In_4_Se_2.25_ had anisotropic Seebeck properties, but there are no existing studies regarding the effective properties of polycrystals with anisotropic Seebeck properties. Therefore, the comparison on the cooling performances for the In_4_Se_2.25_/Copper composite between composites with isotropic and anisotropic In_4_Se_2.25_ phase properties was not presented.

## 5. Conclusions

This study investigated the transverse thermoelectric properties of fibrous composites with anisotropic component materials. A mathematical model was built for predicting the transverse thermoelectric figure of merit (Z_trans_T) and maximum cooling capacity (∆T_max_) of the composite. The effectiveness of the mathematical model was verified by finite element simulations. The agreement among the two approaches indicated that the mathematical model can serve as an efficient tool for selecting and screening prospecting candidates for fibrous transverse thermoelectric composites with anisotropic material components. A case study using anisotropic Bi_2_Te_3_ polycrystal as the matrix and isotropic Copper as the fiber was performed and a ∆T_max_ of 34 K was reached. 

The variance in the maximum Z_trans_T and ∆T_max_ values for both the layered and fibrous transverse thermoelectric composites was investigated with respect to the possible anisotropic properties of component phase. The results showed that by enhancing the anisotropic profile of polycrystal, the maximum Z_trans_T and ∆T_max_ for both the layered and fibrous transverse thermoelectric composites can be improved. For the SnSe/Copper composite at 700 K, the improvement of maximum Z_trans_T and ∆T_max_ values for the composite with anisotropic SnSe single-crystal components can be as much as 51% compared to the composites with isotropic SnSe polycrystals. 

This study also showed that the alignment of the component’s local property axes in the composite can lead to significant variance in the Z_trans_T and ∆T_max_ values of the composite. For the Bi_2_Te_3_/Copper composite, the difference in the thermoelectric performance caused by the alignment of the anisotropic component’s property axis was 50%. For the SnSe/Copper composite, this difference exceeded 400%. This discrepancy was mainly caused by the variance in the anisotropic properties among different material property axis directions in the component phase.

## Figures and Tables

**Table 1:** Material properties used in this study.

Material	Single Crystal Bi_2_Te_3_	Polycrystal Bi_2_Te_3_	Single Crystal In_4_Se_2.25_	Single Crystal SnSe	Copper	Copper	Copper
Measuring Temperature	300 K	300 K	600 K	700 K	300 K	600 K	700 K
*S_w_* (μV/K)	−210	−187	−375	−540	2.83	3.34	3.84
*S_u_*/*S_v_* (μV/K)			−313				
*ρ_w_* (Ω·m)	4.5 × 10^−5^	2.4 × 10^−5^	2.0 × 10^−4^	1.0 × 10^−2^	1.7 × 10^−8^	4.0 × 10^−8^	5.0 × 10^−8^
*ρ_u_*\*ρ_v_* (Ω·m)	1.5 × 10^−5^	1.0 × 10^−5^	1.0 × 10^−4^	1.1 × 10^−3^			
*λ_w_* (W·m^−1^·K^−1^)	1.00	0.78	0.80	0.25	400	386	377
*λ_u_*\*λ_v_* (W·m^−1^·K^−1^)	1.55	1.17	1.15	0.35			
Reference	[17]	[5]	[29]	[18]	[30,31]	[30,31]	[30,31]

*T*: Temperature; *S*: Seebeck coefficient; *ρ*: electrical resistivity; *λ*: thermal conductivity.

**Table 2:** Comparison of ∆T_max_ between the mathematical model and the finite element simulation model.

Volume Fraction of Copper	Configuration I			Configuration II		
	Mathematical Model (K)	Square Fiber (K)	Cylindrical Fiber (K)	Mathematical Model (K)	Square Fiber (K)	Cylindrical Fiber (K)
0.1	32.59	29.95	25.67	23.28	20.74	21.37
0.2	30.63	28.76	28.39	19.69	19.31	18.91
0.3	29.03	26.59	27.36	17.93	18.02	17.86
0.4	28.40	26.41	27.25	17.33	18.41	18.22
0.5	28.57	28.16	27.98	17.55	19.04	19.22

**Table 3:** ∆T_max_ and maximum Z_trans_T for different types of transverse thermoelectric composites.

Component 1		Bi_2_Te_3_ Single Crystal		In_4_Se_2.25_ Single Crystal		SnSe Single Crystal	
Component 2		Copper					
Operating Temperature		300 K		600 K		700 K	
-		∆T_max_	Z_trans_T	∆T_max_	Z_trans_T	∆T_max_	Z_trans_T
Fibrous Composite	Configuration I	30	0.24	79	0.35	92	0.35
	Configuration II	20	0.16	65	0.28	22	0.07
	Average RV (Reuss and Voigt models)	26	0.21	N/A	N/A	61	0.21
Layered Composite	Configuration III	20	0.16	65	0.28	22	0.07
	Configuration IV	30	0.24	80	0.35	92	0.35
	Average RV	26	0.21	N/A	N/A	61	0.21
